# Supplementary material for: APTC-EC-2A: A Lytic Phage Targeting Multidrug Resistant E. coli Planktonic Cells and Biofilms
Source: Microorganisms. 2022 Jan 4;10(1):102. doi: 10.3390/microorganisms10010102 (PMC8779906; doi:10.3390/microorganisms10010102)
Supplement: Supplementary file 1 [file microorganisms-10-00102-s001.zip › microorganisms-1502434-supplementary.pdf]

Supplementary:

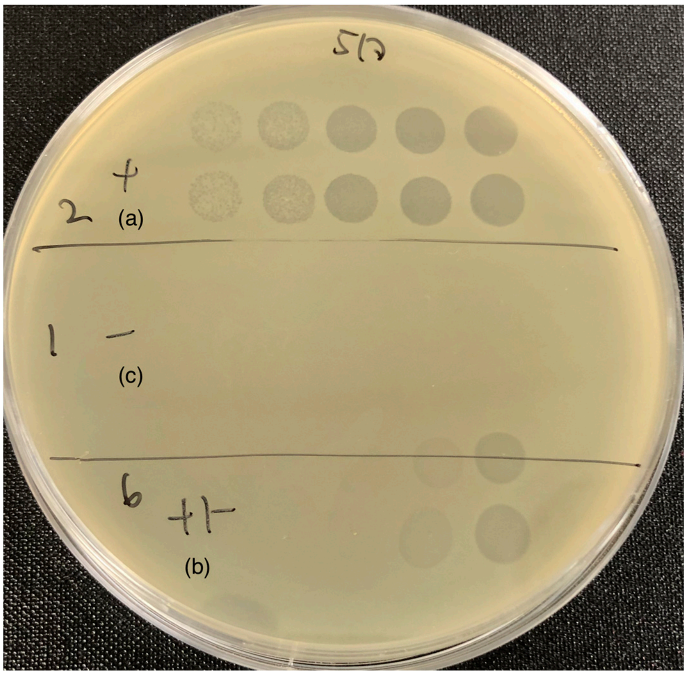

Figure S1. Susceptibility of *E. coli* to phage lysis. Representative LB agar plate showing the three categories of phage spots: (a) clear, (b) turbid and (c) no reaction; denoting sensitive, semi-sensitive and resistance to phage respectively.

Table S1. The annotation information, such as position, direction and functions of each gene and conserved proteins.

Type	Start	End	Strand	Attributes
CDS	1	2,178	-	CDS_1_rIIA lysis inhibitor
CDS	2,189	2,392	-	CDS_2_topoisomerase
CDS	2,447	4,264	-	CDS_3_DNA gyrase subunit B
CDS	4,203	4,607	+	CDS_4_hypothetical protein
CDS	4,600	4,971	-	CDS_5_hypothetical protein
CDS	4,974	5,150	-	CDS_6_hypothetical protein
CDS	5,153	5,566	-	CDS_7_mRNA metabolism modulator goF
CDS	5,566	5,781	-	CDS_8_modifier of suppressor tRNAs
CDS	5,799	5,888	+	CDS_9_Transcription regulatory protein motB
CDS	5,954	6,445	-	CDS_10_modifier of transcription
CDS	6,522	7,067	-	CDS_11_transcription regulator MotB domain-containing protein
CDS	7,070	7,570	-	CDS_12_exodeoxyribonuclease

CDS	7,634	8,317	-	CDS_13_exonuclease A
CDS	8,317	8,559	-	CDS_14_hypothetical protein
CDS	8,552	8,797	-	CDS_15_ATP-dependent DNA helicase dda
CDS	8,784	9,044	-	CDS_16_hypothetical protein
CDS	9,051	10,370	-	CDS_17_hypothetical protein
CDS	10,367	10,678	-	CDS_18_hypothetical protein
CDS	10,680	11,426	-	CDS_19_ADP-ribosyltransferase modB
CDS	11,549	12,151	-	CDS_20_ADP-ribosyltransferase modB
CDS	12,148	12,771	-	CDS_21_ADP-ribosylase
CDS	12,839	13,021	-	CDS_22_hypothetical protein
CDS	13,030	13,500	-	CDS_23_hypothetical protein
CDS	13,493	13,645	-	CDS_24_molybdenum ABC transporter
CDS	13,654	13,839	-	CDS_25_transcription modulator
CDS	13,832	14,317	-	CDS_26_transcription modulator under heat shock
CDS	14,326	14,667	-	CDS_27_hypothetical protein
CDS	14,667	14,873	-	CDS_28_small outer capsid protein
CDS	14,854	14,976	-	CDS_29_small outer capsid protein
CDS	14,969	15,214	-	CDS_30_capsid and scaffold protein
CDS	15,231	15,440	-	CDS_31_small outer capsid protein
CDS	15,437	15,631	-	CDS_32_small outer capsid protein
CDS	15,631	16,149	-	CDS_33_dCTP pyrophosphatase
CDS	16,210	16,989	-	CDS_34_DNA adenine methyltransferase
CDS	16,989	17,549	-	CDS_35_hypothetical protein
CDS	17,568	17,657	-	CDS_36_hypothetical protein
CDS	17,649	17,849	+	CDS_37_DNA primase
CDS	17,846	18,874	-	CDS_38_putative DNA primase catalytic core
CDS	18,877	19,041	-	CDS_39_hypothetical protein
CDS	19,043	19,399	-	CDS_40_hypothetical protein
CDS	19,401	20,027	-	CDS_41_spackle protein
CDS	20,027	20,320	-	CDS_42_spackle periplasmic protein
CDS	20,379	20,636	-	CDS_43_hypothetical protein
CDS	20,638	20,820	-	CDS_44_discriminator of mRNA degradation
CDS	20,879	22,306	-	CDS_45_replicative DNA helicase
CDS	22,316	22,660	-	CDS_46_head vertex assembly chaperone
CDS	22,653	23,834	-	CDS_47_RecA-like recombination protein

CDS	23,912	24,754	-	CDS_48_thymidylate synthase
CDS	24,751	25,491	-	CDS_49_deoxycytidylate 5-hydroxymethyltransferase
CDS	25,499	25,648	-	CDS_50_hypothetical protein
CDS	25,645	25,896	-	CDS_51_immunity to superinfection membrane protein
CDS	25,904	26,284	-	CDS_52_hypothetical protein
CDS	26,295	26,531	-	CDS_53_DNA polymerase
CDS	26,714	29,410	-	CDS_54_DNA polymerase
CDS	29,489	29,857	-	CDS_55_translational repressor protein
CDS	29,859	30,422	-	CDS_56_clamp-loader subunit
CDS	30,424	31,383	-	CDS_57_clamp loader subunit DNA polymerase accessory protein
CDS	31,435	32,121	-	CDS_58_sliding clamp DNA polymerase accessory protein
CDS	32,177	32,566	-	CDS_59_RNA polymerase binding protein
CDS	32,576	32,764	-	CDS_60_exonuclease subunit
CDS	32,820	34,460	-	CDS_61_recombination endonuclease subunit
CDS	34,499	34,714	-	CDS_62_hypothetical protein
CDS	34,686	34,949	-	CDS_63_recombination endonuclease subunit
CDS	34,946	35,965	-	CDS_64_recombination endonuclease subunit
CDS	35,962	36,102	-	CDS_65_DNA alpha-glucosyltransferase
CDS	36,142	37,344	-	CDS_66_DNA alpha-glucosyltransferase
CDS	37,411	37,524	-	CDS_67_hypothetical protein
CDS	37,588	37,791	-	CDS_68_hypothetical protein
CDS	37,760	38,077	-	CDS_69_hypothetical protein
CDS	38,079	38,297	-	CDS_70_hypothetical protein
CDS	38,281	38,838	-	CDS_71_RNA polymerase sigma factor
CDS	38,917	39,186	-	CDS_72_hypothetical protein
CDS	39,183	39,398	-	CDS_73_hypothetical protein
CDS	39,401	39,727	-	CDS_74_hypothetical protein
CDS	39,780	39,980	-	CDS_75_hypothetical protein
CDS	39,981	40,112	-	CDS_76_hypothetical protein
CDS	40,120	40,413	-	CDS_77_hypothetical protein
CDS	40,406	40,588	-	CDS_78_hypothetical protein
CDS	40,747	41,055	-	CDS_79_glutaredoxin

CDS	41,058	41,270	-	CDS_80_anaerobic ribonucleoside-triphosphate reductase-activating protein
CDS	41,279	41,371	-	CDS_81_anaerobic ribonucleoside-triphosphate reductase-activating protein
CDS	41,385	41,855	-	CDS_82_Pyruvate formate-lyase 1-activating enzyme
CDS	41,852	43,669	-	CDS_83_anaerobic ribonucleoside-triphosphate reductase-activating protein
CDS	43,666	44,139	-	CDS_84_packaging and recombination endonuclease VII
CDS	44,181	44,357	-	CDS_85_host protease inhibitor
CDS	44,394	44,879	-	CDS_86_host protease inhibitor
CDS	44,863	45,030	-	CDS_87_hypothetical protein
CDS	45,002	45,172	-	CDS_88_hypothetical protein
CDS	45,175	45,390	-	CDS_89_glutaredoxin
CDS	45,387	45,650	-	CDS_90_glutaredoxin
CDS	45,652	45,894	-	CDS_91_thioredoxin
CDS	45,881	46,195	-	CDS_92_thioredoxin
CDS	46,192	47,121	-	CDS_93_thioredoxin
CDS	47,174	48,175	-	CDS_94_thioredoxin
CDS	48,233	49,255	-	CDS_95_thioredoxin
CDS	49,264	50,151	-	CDS_96_thioredoxin
CDS	50,163	50,567	-	CDS_97_thioredoxin
CDS	50,623	51,150	-	CDS_98_thioredoxin
CDS	51,211	51,513	-	CDS_99_thioredoxin
CDS	51,615	52,583	-	CDS_100_thioredoxin
CDS	52,699	53,712	-	CDS_101_hypothetical protein
CDS	53,709	54,170	-	CDS_102_hypothetical protein
CDS	54,173	54,694	-	CDS_103_hypothetical protein
CDS	54,701	55,234	-	CDS_104_hypothetical protein
CDS	55,261	55,365	-	CDS_105_hypothetical protein
CDS	55,426	55,599	-	CDS_106_hypothetical protein
CDS	55,589	55,783	-	CDS_107_hypothetical protein
CDS	55,786	55,989	-	CDS_108_hypothetical protein
CDS	55,989	56,177	-	CDS_109_hypothetical protein
CDS	56,272	56,658	-	CDS_110_antiholin

CDS	56,655	56,948	-	CDS_111_rI lysis inhibition regulator
CDS	56,961	57,173	-	CDS_112_thymidine kinase
CDS	57,216	57,797	-	CDS_113_thymidine kinase
CDS	57,807	57,992	-	CDS_114_hypothetical protein
CDS	57,989	58,162	-	CDS_115_hypothetical protein
CDS	58,159	58,371	-	CDS_116_hypothetical protein
CDS	58,362	58,574	-	CDS_117_hypothetical protein
CDS	58,571	59,038	-	CDS_118_valyl–tRNA ligase modifier
CDS	59,035	59,376	-	CDS_119_valyl-tRNA synthetase modifier
CDS	59,369	59,914	-	CDS_120_endoribonuclease RegB domain-containing protein
CDS	59,922	60,383	-	CDS_121_endoribonuclease RegB domain-containing protein
CDS	60,443	60,721	-	CDS_122_endoribonuclease RegB domain-containing protein
CDS	60,721	60,987	-	CDS_123_hypothetical protein
CDS	60,980	61,201	-	CDS_124_autonomous glycyl radical cofactor
CDS	61,201	61,563	-	CDS_125_autonomous glycyl radical cofactor
CDS	61,570	61,899	-	CDS_126_endoribonuclease RegB domain-containing protein
CDS	61,896	62,465	-	CDS_127_endoribonuclease RegB domain-containing protein
CDS	62,581	62,997	-	CDS_128_endonuclease
CDS	63,057	63,551	-	CDS_129_lysozyme murein hydrolase e
CDS	63,588	64,043	-	CDS_130_nudix hydrolase
CDS	64,025	64,513	-	CDS_131_hypothetical protein
CDS	64,510	64,872	-	CDS_132_hypothetical protein
CDS	64,854	65,246	-	CDS_133_hypothetical protein
CDS	65,215	65,829	-	CDS_134_hypothetical protein
CDS	65,871	66,464	-	CDS_135_hypothetical protein
CDS	66,566	66,829	-	CDS_136_hypothetical protein
CDS	66,843	66,953	+	CDS_137_hypothetical protein
CDS	67,059	67,622	-	CDS_138_hypothetical protein
CDS	67,656	67,751	-	CDS_139_hypothetical protein
CDS	67,748	68,221	-	CDS_140_hypothetical protein
CDS	68,404	68,532	-	CDS_141_hypothetical protein

CDS	68,605	68,955	-	CDS_142_hypothetical protein
CDS	70,089	70,376	-	CDS_143_hypothetical protein
CDS	70,379	70,777	-	CDS_144_hypothetical protein
CDS	70,777	70,962	-	CDS_145_hypothetical protein
CDS	71,021	71,266	-	CDS_146_hypothetical protein
CDS	71,331	71,588	-	CDS_147_hypothetical protein
CDS	71,662	72,120	-	CDS_148_hypothetical protein
CDS	72,117	72,347	-	CDS_149_tail fiber assembly protein
CDS	72,347	73,072	-	CDS_150_deoxynucleotide monophosphate kinase
CDS	73,122	73,652	-	CDS_151_tail completion and sheath stabilizer protein
CDS	73,759	74,418	-	CDS_152_putative site-specific intron-like DNA endonuclease
CDS	74,415	75,242	-	CDS_153_putative DNA end protector protein
CDS	75,242	75,694	-	CDS_154_head completion protein
CDS	75,742	76,332	+	CDS_155_baseplate central spike complex protein
CDS	76,316	78,043	+	CDS_156_baseplate hub + tail lysozyme
CDS	78,018	78,572	+	CDS_157_baseplate puncturing device
CDS	78,573	78,866	+	CDS_158_baseplate wedge protein
CDS	78,875	80,857	+	CDS_159_baseplate wedge subunit
CDS	80,854	83,952	+	CDS_160_baseplate wedge subunit
CDS	83,945	84,949	+	CDS_161_baseplate wedge subunit
CDS	85,013	85,879	+	CDS_162_baseplate wedge tail fiber connector
CDS	85,879	87,684	+	CDS_163_baseplate wedge subunit and tail pin
CDS	87,684	88,343	+	CDS_164_baseplate wedge subunit and tail pin
CDS	88,340	89,797	+	CDS_165_short tail fiber
CDS	89,801	89,890	+	CDS_166_short tail fiber protein
CDS	89,900	91,357	+	CDS_167_fibritin neck whiskers
CDS	91,389	92,318	+	CDS_168_head completion, neck hetero-dimeric protein
CDS	92,320	93,090	+	CDS_169_head completion, neck hetero-dimeric protein

CDS	93,132	93,950	+	CDS_170_tail sheath stabilizer and completion protein
CDS	93,959	94,453	+	CDS_171_terminase DNA packaging enzyme small subunit
CDS	94,437	96,269	+	CDS_172_terminase DNA packaging enzyme large subunit
CDS	96,301	98,280	+	CDS_173_putative long tail fiber proximal subunit
CDS	98,397	98,888	+	CDS_174_tail tube protein
CDS	98,972	100,546	+	CDS_175_portal vertex protein of head
CDS	100,546	100,785	+	CDS_176_prohead core protein
CDS	100,785	101,210	+	CDS_177_prohead core protein
CDS	101,210	101,848	+	CDS_178_prohead core scaffold protein and protease
CDS	101,879	102,688	+	CDS_179_prohead core scaffold protein
CDS	102,707	104,272	+	CDS_180_putative major capsid protein
CDS	104,356	105,639	+	CDS_181_putative precursor of head vertex subunit
CDS	105,669	106,673	-	CDS_182_RNA ligase
CDS	106,683	106,961	-	CDS_183_hypothetical protein
CDS	106,948	107,163	-	CDS_184_highly immunogenic outer capsid protein
CDS	107,253	108,383	-	CDS_185_large head outer capsid protein
CDS	108,393	109,073	-	CDS_186_ATP-dependent DNA helicase uvsW
CDS	109,124	110,635	+	CDS_187_ATP-dependent DNA helicase uvsW
CDS	110,661	110,891	+	CDS_188_RNA-DNA and DNA-DNA helicase ATPase
CDS	110,947	111,114	-	CDS_189_hypothetical protein
CDS	111,143	111,367	-	CDS_190_recombination protein uvsY
CDS	111,367	111,780	-	CDS_191_recombination, repair and ssDNA binding protein
CDS	111,847	112,245	-	CDS_192_baseplate wedge subunit
CDS	112,245	112,871	-	CDS_193_putative baseplate hub subunit
CDS	112,922	113,674	+	CDS_194_baseplate hub assembly protein
CDS	113,671	114,843	+	CDS_195_base plate hub subunit

CDS	114,791	115,324	+	CDS_196_baseplate distal hub subunit
CDS	115,321	117,093	+	CDS_197_base plate hub
CDS	117,102	118,196	+	CDS_198_baseplate tail tube cap
CDS	118,196	119,161	+	CDS_199_base plate-tail tube initiator
CDS	119,190	119,480	-	CDS_200_NAD(+)–arginine ADP-ribosyltransferase
CDS	119,541	121,598	-	CDS_201_NAD(+)–arginine ADP-ribosyltransferase
CDS	121,602	123,650	-	CDS_202_Alt RNA polymerase ADP-ribosylase
CDS	123,703	123,891	-	CDS_203_DNA ligase
CDS	123,888	125,351	-	CDS_204_DNA ligase
CDS	125,348	125,617	-	CDS_205_hypothetical protein
CDS	125,617	126,456	-	CDS_206_hypothetical protein
CDS	126,453	126,911	-	CDS_207_hypothetical protein
CDS	126,904	127,110	-	CDS_208_hypothetical protein
CDS	127,107	127,304	-	CDS_209_hypothetical protein
CDS	127,304	127,591	-	CDS_210_hypothetical protein
CDS	127,632	127,997	-	CDS_211_hypothetical protein
CDS	128,065	128,397	-	CDS_212_hypothetical protein
CDS	128,508	128,726	-	CDS_213_lysis inhibition accessory protein
CDS	128,717	128,827	-	CDS_214_protein rIII
CDS	128,832	129,014	-	CDS_215_lysis inhibition accessory protein
CDS	129,083	129,331	-	CDS_216_lysis inhibition accessory protein
CDS	129,479	129,814	-	CDS_217_head assembly cochaperone with GroEL
CDS	129,871	130,179	-	CDS_218_tail fiber protein
CDS	130,180	130,416	-	CDS_219_deoxycytidylate deaminase
CDS	130,416	130,997	-	CDS_220_dCMP deaminase
CDS	130,994	131,332	-	CDS_221_hypothetical protein
CDS	131,329	131,565	-	CDS_222_hypothetical protein
CDS	131,559	132,086	-	CDS_223_hypothetical protein
CDS	132,149	132,424	-	CDS_224_hypothetical protein
CDS	132,427	132,627	-	CDS_225_hypothetical protein
CDS	132,620	132,796	-	CDS_226_hypothetical protein

CDS	132,816	133,721	-	CDS_227_3′-phosphatase, 5′-polynucleotide kinase
CDS	133,718	134,038	-	CDS_228_hypothetical protein
CDS	134,035	134,265	-	CDS_229_hypothetical protein
CDS	134,262	134,561	-	CDS_230_Spanin Rz1
CDS	134,558	134,911	-	CDS_231_Spanin Rz
CDS	134,902	135,408	-	CDS_232_host transcription inhibitor
CDS	135,470	136,594	-	CDS_233_RNA ligase
CDS	136,647	137,057	-	CDS_234_endonuclease
CDS	137,085	138,263	-	CDS_235_ribonucleoside-diphosphate reductase subunit beta
CDS	138,315	140,579	-	CDS_236_ribonucleoside-diphosphate reductase subunit alpha
CDS	140,570	140,896	-	CDS_237_hypothetical protein
CDS	140,850	141,113	-	CDS_238_thymidylate synthase
CDS	141,110	141,970	-	CDS_239_thymidylate synthase
CDS	142,016	142,363	-	CDS_240_dihydrofolate reductase
CDS	142,383	142,964	-	CDS_241_dihydrofolate reductase
CDS	142,964	143,209	-	CDS_242_hypothetical protein
CDS	143,220	143,462	-	CDS_243_hypothetical protein
CDS	143,601	143,966	-	CDS_244_hypothetical protein
CDS	144,011	144,238	-	CDS_245_hypothetical protein
CDS	144,252	144,362	-	CDS_246_hypothetical protein
CDS	144,383	145,291	-	CDS_247_ssDNA binding, DNA repair, recombination and pre-synthesis protein
CDS	145,391	146,029	-	CDS_248_DNA helicase loader
CDS	146,041	146,379	-	CDS_249_late promoter transcription accessory protein
CDS	146,357	146,626	-	CDS_250_dsDNA binding protein
CDS	146,635	147,552	-	CDS_251_DNA polymerase I
CDS	147,657	151,526	+	CDS_252_proximal tail fiber subunit
CDS	151,535	152,650	+	CDS_253_hinge connector of long tail fiber proximal connector
CDS	152,713	153,363	+	CDS_254_hinge connector long tail fiber
CDS	153,372	156,317	+	CDS_255_large distal tail fiber subunit
CDS	156,354	157,136	+	CDS_256_tail fiber

CDS	157,167	157,823	+	CDS_257_holin
CDS	157,824	158,096	-	CDS_258_anti-sigma factor
CDS	158,109	158,261	-	CDS_259_anti-restriction endonuclease
CDS	158,258	158,536	-	CDS_260_inhibitor of MrcBC restriction endonuclease
CDS	158,620	158,751	-	CDS_261_hypothetical protein
CDS	158,822	159,118	-	CDS_262_anti-restriction nuclease
CDS	159,118	159,585	-	CDS_263_hypothetical protein
CDS	159,582	159,737	-	CDS_264_putative baseplate wedge initiator
CDS	159,734	160,063	-	CDS_265_middle transcription regulatory protein motA
CDS	160,074	160,709	-	CDS_266_activator of middle period transcription
CDS	160,815	161,042	-	CDS_267_hypothetical protein
CDS	161,178	161,327	-	CDS_268_topoisomerase
CDS	161,324	162,652	-	CDS_269_DNA topoisomerase II medium subunit
CDS	162,657	162,797	-	CDS_270_hypothetical protein
CDS	162,790	162,948	-	CDS_271_acridine resistance protein
CDS	163,036	163,494	-	CDS_272_nucleoid disruption protein
CDS	163,555	163,770	-	CDS_273_hypothetical protein
CDS	163,779	163,886	-	CDS_274_hypothetical protein
CDS	163,886	164,083	-	CDS_275_hypothetical protein
CDS	164,091	164,204	-	CDS_276_inner membrane protein
CDS	164,270	164,368	-	CDS_277_outer membrane protein
CDS	164,448	164,711	-	CDS_278_hypothetical protein
CDS	164,792	165,349	-	CDS_279_endonuclease
CDS	165,436	165,630	-	CDS_280_hypothetical protein
CDS	165,659	166,597	-	CDS_281_rIIB lysis inhibitor
trna	68,974	69,049	-	tRNA-Arg(TCT)
trna	69,054	69,129	-	tRNA-His(GTG)
trna	69,244	69,318	-	tRNA-Asn(GTT)
trna	69,323	69,409	-	tRNA-Tyr(GTA)
trna	69,419	69,493	-	tRNA-Ile2(CAT)
trna	69,495	69,570	-	tRNA-Thr(TGT)
trna	69,576	69,665	-	tRNA-Ser(TGA)

trna	69,668	69,741	-	tRNA-Pro(TGG)
trna	69,752	69,825	-	tRNA-Gly(TCC)
trna	69,831	69,917	-	tRNA-Leu(TAA)
trna	69,919	69,991	-	tRNA-Gln(TTG)
